# Integrated transcriptome and metabolome analyses uncover stage-specific dynamic regulatory networks during sesame floral development

**DOI:** 10.3389/fpls.2026.1918255

**Published:** 2026-08-13

**Authors:** Qiyuan An, Hongsen Cheng, Huijie Sun, Yanbin Na, Guangzhen Zhou, Dexue Gao

**Affiliations:** 1 Liaoning Research Institute of Cash Crops, Liaoyang, Liaoning, China; 2 Ministry of Education Key Laboratory for Ecology of Tropical Islands, Key Laboratory of Tropical Animal and Plant Ecology of Hainan Province, College of Life Sciences, Hainan Normal University, Haikou, China

**Keywords:** flavonoid, floral development, metabolome, phenylpropanoid pathway, *Sesamum indicum* L., stage-specific expression, transcriptome, WGCNA

## Abstract

Sesame is an important oilseed crop, and floral development is a key biological process that lays the foundation for pollination, fertilization, and seed formation, which are closely associated with final yield potential. However, the dynamic transcriptional and metabolic regulatory mechanisms during floral development remain unclear. Here, we performed an integrated transcriptomic and metabolomic analysis across five key developmental stages (T1–T5) of sesame flowers to systematically dissect the multi‑omics regulatory network. KEGG enrichment analysis revealed distinct stage‑specific metabolic characteristics: early stages (T1–T2) were enriched in primary energy metabolism (glycolysis and starch/sucrose metabolism); the middle stage (T3) showed enrichment in DNA replication and phenylpropanoid biosynthesis; and late stages (T4–T5) were associated with plant hormone signaling and α‑linolenic acid metabolism. WGCNA identified two modules correlated with development: a positive module involved in phenylpropanoid biosynthesis, and a negative module related to DNA replication and repair. Genes in the phenylpropanoid/flavonoid pathway displayed a clear sequential expression pattern, promoting flavonoid and anthocyanin accumulation. Collectively, this study provides a comprehensive multi‑omics resource and a descriptive framework for understanding transcriptional and metabolic dynamics during sesame floral development, and identifies candidate pathways and genes that may serve as targets for future functional validation and molecular breeding.

## 1 Introduction

Floral development is a highly coordinated and dynamic process that proceeds from floral primordium initiation, through organ differentiation, to anthesis. This process is precisely regulated by the sophisticated crosstalk among genetic, epigenetic, and environmental regulatory networks (Ó’Maoiléidigh et al., 2014; Wellmer and Riechmann, 2010). A key transition during the plant life cycle is the shift from vegetative to reproductive growth, which is controlled by core transcription factors including LEAFY (*LFY*) and APETALA1 (*AP1*) (Ó’Maoiléidigh et al., 2014; Siriwardana and Lamb, 2012) [1,3]. Floral organ identity and patterning follow the conserved ABCDE model, in which MADS-domain transcription factors act combinatorially to specify sepals, petals, stamens, and carpels (Kim et al., 2005; Theissen et al., 2002). Although these central regulators are evolutionarily conserved, the metabolic pathways they modulate often exhibit distinct species-specific characteristics (Kim et al., 2005; Theissen et al., 2002) 36. In addition to transcription factor genes, genes involved in hormone biosynthesis, transport, and signaling are also over-represented among the targets of floral organ identity genes. For instance, auxin signaling plays critical roles in floral meristem patterning, the growth of floral organ primordia, and multiple morphogenetic processes during floral organogenesis (Cheng and Zhao, 2007; Stewart et al., 2016). Other major plant hormones, including cytokinins, gibberellins, and jasmonic acid, also contribute to the regulation of floral development (Bartrina et al., 2011; Chandler, 2011). Concurrently, global metabolic reprogramming occurs during floral development. Dynamic changes in sugars and amino acids provide essential energy and carbon skeletons for cell proliferation and growth, while secondary metabolites such as flavonoids participate in floral pigmentation, defense responses, and stress tolerance (Bellaire et al., 2014; Adebesin et al., 2017; Borghi et al., 2017; Fornoff et al., 2017). The integrated regulatory network formed by the crosstalk among floral developmental regulators, hormone pathways, and metabolic dynamics ensures the robust and accurate progression of floral development.

Sesame (*Sesamum indicum* L.) is a globally cultivated oilseed prized for its nutrient-rich seeds (Dossa et al., 2018; Miao et al., 2021). Its yield is closely tied to successful floral development, which follows a specific pattern: racemose inflorescences bearing flowers in leaf axils of the main stem or branches (Mei et al., 2017; Sabag et al., 2021). Its floral development process includes the formation of inflorescence primordia, bract primordia, and terminal floral primordia. Although the regulatory network of floral development has been well studied in model plants, the molecular and metabolic mechanisms underlying floral developmental dynamics in sesame remain largely unclear. As a typical short-day plant, sesame flowering is strongly promoted under short-day conditions. Flowering time is an important agronomic trait that affects environmental adaptation (Zhou et al., 2018). Genetic analyses have demonstrated that flowering time in sesame is a quantitative trait regulated by photoperiod, and homologs of CONSTANS (*SiCOL1*) participate in this process (Zhou et al., 2018). Several key QTLs for flowering time have been identified via genome-wide association studies across diverse environments (Sabag et al., 2021). In addition to flowering transition, the precise regulation of floral organ identity and structure is essential for subsequent reproductive processes. For example, loss of function of the *CEN2* (centroradialis-like) gene increases flower number per leaf axil, indicating its key role in controlling floral meristem activity (Mei et al., 2017; Song et al., 2023). However, previous studies on sesame floral development have relied predominantly on morphological observations or functional characterization of individual genes/QTLs, and a time-resolved multi-omics dataset covering the entire floral development process is still lacking. Although individual regulators such as SiCOL1 and CEN2 have been identified, these studies provide only a fragmented view of the regulatory landscape, as they do not capture the global transcriptional and metabolic reprogramming that occurs dynamically across sequential developmental stages. Without systematic multi-omics profiling, the coordinated regulatory networks governing the progression from floral initiation to organ maturation remain largely unexplored. Furthermore, the phenylpropanoid–flavonoid pathway, which is well known for its critical roles in pigmentation, stress responses, and reproductive development in model plants (Dixon and Steele, 1999; Fraser and Chapple, 2011; Winkel-Shirley, 2001), has not been characterized in the context of sesame floral organs. Although flavonoids have been shown to be indispensable for male fertility in rice (Wang et al., 2020) and contribute to floral pigmentation and pollinator attraction in diverse species (Borghi et al., 2017; Saigo et al., 2020), whether and how this pathway temporally regulates floral organ maturation and reproductive success in sesame remains completely unknown. Moreover, sesame is a typical short-day plant, yet the potential crosstalk between photoperiod signaling and secondary metabolic networks during floral development has not been explored. While the photoperiod-responsive gene SiCOL1 has been identified as a regulator of flowering time in sesame (Zhou et al., 2018), it remains unknown whether photoperiod signals directly or indirectly influence the accumulation of phenylpropanoid/flavonoid metabolites and the expression of related biosynthetic genes during floral development. Given that photoperiod is a key environmental cue that affects both flowering transition and secondary metabolism in many plant species, this represents a significant knowledge gap that requires urgent attention.

In recent years, integrative multi-omics approaches have been increasingly applied to dissect the molecular mechanisms underlying floral development and floral trait variation in other oilseed crops. In rapeseed (*Brassica napus*), combined transcriptomic and metabolomic analyses have successfully uncovered the regulatory networks governing anthocyanin-mediated flower color variation across multiple genotypes and developmental stages, identifying key structural genes (e.g., *CHS*, *F3H*, *UGT*) and transcription factors (e.g., *MYB75*, *MYB12*, *MYB111*, *GL3*, *TTG1*) involved in pigment accumulation via weighted gene co-expression network analysis (Cui et al., 2024). Similarly, integrated multi-omics and co-expression network analyses of orange-red petal formation in *B. napus* revealed that 51 differentially expressed genes were linked to anthocyanin metabolism, including structural genes such as *BnaCHS*, *BnaF3H*, *BnaDFR*, *BnaANS*, *BnaUGTs*, and transcription factor genes including *BnaHY5*, *BnaPAP2*, *BnaTT8*, and *BnaTTG2* (Jia et al., 2025). In peanut (*Arachis hypogaea*), a comprehensive multi-omics platform integrating transcriptomic, proteomic, and metabolomic data across 22 tissues has been established, providing a valuable resource for understanding gene and metabolite regulation throughout reproductive development (Xue et al., 2026). In soybean (*Glycine max*), integrated transcriptomic and metabolomic analyses have revealed developmental stage-specific molecular responses during flowering, identifying stage-specific gene expression and metabolic reprogramming patterns associated with floral transition and reproductive development (Liu et al., 2025). These studies collectively demonstrate the power of multi-omics integration in dissecting floral developmental processes and reproductive traits in oilseed crops. However, despite the agronomic and economic importance of sesame (*Sesamum indicum* L.), a time-resolved multi-omics characterization covering the entire floral development process has remained unavailable, and the regulatory roles of key metabolic pathways—particularly the phenylpropanoid/flavonoid pathway—in sesame floral organs remain largely uncharacterized.

To address the current gap, we investigated the transcriptional and metabolic dynamics across different developmental stages, with a particular focus on how key metabolic pathways (especially the phenylpropanoid/flavonoid pathway) temporally regulate floral organ maturation. We performed integrated transcriptomic and metabolomic profiling across five key developmental stages (T1–T5) in the sesame cultivar ‘Liaozhi No. 8’. Through differential expression analysis, temporal clustering, co-expression network construction, pathway enrichment, and transcript-metabolite correlation, our study elucidates the core regulatory network and pivotal metabolic pathways underpinning sesame floral development, with focused investigation into the phenylpropanoid/flavonoid biosynthetic pathway. These findings provide novel insights into the molecular and metabolic basis of floral development in sesame and offer valuable gene resources for future research on floral development regulation and reproductive biology in sesame.

## 2 Materials and methods

### 2.1 Plant materials and sampling

The sesame cultivar ‘Liaozhi No. 8’ was used in this study. Plants were grown in a greenhouse at the Cash Crops Research Institute, Liaoning Province, under natural light conditions. Environmental parameters during the sampling period were as follows: temperature 25–28°C, relative humidity 60–70%, natural photoperiod (approximately 14 h light/10 h dark), which corresponds to the local summer daylight length (May–August). Although sesame is classified as a short-day plant, the local cultivar used here has been long cultivated in this region and is well adapted to this photoperiod; phenotypic monitoring confirmed normal flowering and fruiting without obvious developmental delays or abnormalities under these conditions. Floral buds and flowers were collected at five developmental stages: floral bud formation stage (T1, 3 days after bud initiation), bud maturation stage (T2, 7 days), petal expansion stage (T3, 10 days), stamen maturation stage (T4, 15 days), and pistil maturation stage (T5, 18 days). All samples were collected between 9:00–10:00 AM to minimize diurnal variation. Three independent biological replicates were obtained from individual plants. Collected samples were immediately frozen in liquid nitrogen and stored at –80°C until further use.

### 2.2 RNA-seq and transcriptome analysis

Total RNA was extracted from floral tissues using the EASYspin Plus Kit (Aidlab, China). RNA integrity was assessed on an Agilent 5300 Bioanalyzer; only samples with RNA Integrity Number (RIN) > 6.5 were used. Stranded cDNA libraries were prepared with the Illumina^®^ Stranded mRNA Prep Kit and sequenced on an Illumina HiSeq X Ten system (Novogene, China) in paired-end mode (150 bp). Raw reads were processed with fastp (version 0.20.0; default parameters) for adapter trimming and quality filtering (minimum Q score ≥ 20) (Chen et al., 2018). The resulting clean reads were aligned to the sesame reference genome GCF_000512975.1 (S_indicum_v1.0) (Wang et al., 2014) using HISAT2 (version 2.2.1; parameters: --rna-strandness RF) (Kim et al., 2015). For each sample, sequencing depth, clean read counts, overall mapping rates, uniquely mapped reads, and gene body coverage were recorded as quality control metrics. Transcript assembly and abundance estimation (FPKM) were performed with StringTie (version 2.1.7; default parameters) (Pertea et al., 2015) for visualization and downstream clustering analyses. Simultaneously, raw gene counts were generated using featureCounts (version 2.0.3) by quantifying reads overlapping with annotated gene models (Liao et al., 2014). Only genes with FPKM > 1 in at least one sample were retained for downstream analyses, and the number of expressed genes per sample was calculated. Raw RNA-seq data were deposited in the NCBI Sequence Read Archive (SRA) under BioProject accession PRJNA1371021; BioSample accession numbers are listed in **Supplementary Table 1**.

### 2.3 Differential gene expression and functional analysis

Differential expression analysis between consecutive stages was performed using DESeq2 (version 1.36.0) with the raw count matrix generated by featureCounts as input, applying thresholds of FDR < 0.05 and |log_2_ fold change| ≥ 1 (Love et al., 2014; Wang et al., 2010). Gene annotations were obtained from NR, Swiss-Prot, KEGG, and GO databases via EggNOG-mapper (version 2.1.9). Homology searches were conducted using DIAMOND (version 2.0.13) with an E-value threshold of 1e^-5^. Functional annotations from multiple databases were integrated by prioritizing Swiss-Prot annotations for GO terms and KEGG orthology assignments, with NR annotations used as supplementary descriptions for unannotated sequences. EggNOG-mapper (version 2.1.9) was employed for Clusters of Orthologous Groups (COG) functional classification. For temporal expression pattern analysis, we used STEM (Short Time-series Expression Miner, version 1.3.13) to cluster genes with similar expression profiles across the five developmental stages (Ernst and Bar Joseph, 2006). GO enrichment analysis was conducted with GOseq (version 1.48.0), and KEGG pathway enrichment with KOBAS (version 3.0.3); both considered FDR < 0.05 as significant (Kanehisa et al., 2016).

### 2.4 Metabolite extraction and LC-MS/MS profiling

For each developmental stage, 100 mg of sesame flower tissue was homogenized in methanol–water (4:1, v/v) solution containing internal standards. The mixture was subjected to cryogenic grinding, low-temperature ultrasonic extraction, and protein precipitation at –20°C, followed by centrifugation at 4°C and 13,000 × g. The supernatant was transferred to chromatographic vials for Liquid Chromatography-Tandem Mass Spectrometry(LC-MS/MS) analysis. A pooled quality control (QC) sample was prepared by mixing equal volumes of extracts from all five stages and injected every 5–10 analytical samples to monitor instrument stability and analytical reproducibility.

Metabolite separation was performed on a UHPLC-Q-Exactive system equipped with a BEH C18 column under gradient elution with water and acetonitrile (both containing 0.1% formic acid) (Chen et al., 2014; Vuppala et al., 2013). Full-scan mass spectrometry data (m/z 50–1200) were acquired in positive and negative ionization modes (Gathungu et al., 2018).

### 2.5 Data processing and metabolic pathway analysis

Raw LC-MS data were processed using Progenesis QI software (version 3.0, Waters Corporation, Milford, USA) for baseline filtering, peak detection, integration, retention time correction, and peak alignment, generating a three-dimensional data matrix containing retention time, mass-to-charge ratio (m/z), and peak intensity. Metabolite identification was performed by searching against public databases, including HMDB (http://www.hmdb.ca/) and Metlin (https://metlin.scripps.edu/), based on accurate mass, retention time, and MS/MS fragmentation pattern matching, with a mass accuracy threshold of < 5 ppm for putative annotation (He et al., 2021; Karaman, 2017). This study employed a non-targeted LC-MS/MS approach for relative quantification; therefore, metabolite abundances are expressed as relative abundance (normalized peak intensities) rather than absolute concentrations (μg/g FW). Putative identifications were assigned based on spectral matching and should be considered tentative in the absence of authentic standards. Data preprocessing steps were as follows: the 80% rule was applied to remove missing values, retaining variables with non-zero values in more than 80% of samples within at least one group; missing values were then imputed with the minimum value of the original matrix; to minimize variations caused by sample preparation and instrument instability, total sum normalization was applied to the peak intensity responses; variables with relative standard deviation (RSD) > 30% in QC samples were removed; and log10 transformation was performed to obtain the final data matrix for subsequent analyses. All differentially accumulated metabolites (DAMs) were identified based on relative abundance data, and all reported accumulation patterns reflect relative changes rather than absolute content differences.

Principal component analysis (PCA) and orthogonal partial least-squares discriminant analysis (OPLS-DA) were performed using the R package ropls (Version 1.6.2) on the preprocessed data matrix, with seven-fold cross-validation to evaluate model stability. Differentially accumulated metabolites (DAMs) were identified based on variable importance in projection (VIP > 1) from the OPLS-DA model and Student’s t-test (p < 0.05). Metabolic pathway annotation of DAMs was performed using the KEGG database (https://www.kegg.jp/kegg/pathway.html), and pathway enrichment analysis was conducted using the Python package scipy.stats, with Fisher’s exact test used to identify the most relevant biological pathways. The raw metabolomics data have been deposited in the National Genomics Data Center (NGDC) under BioProject accession PRJCA065109 (submission numbers OXIM017158–OXIM017162; **Supplementary Table 1**).

### 2.6 Integrated transcriptome and metabolome analysis

For integrated analysis, transcript abundance was expressed as TPM (transcripts per million) for compatibility with metabolomic data, and both transcriptomic and metabolomic datasets were independently normalized using z-score transformation (Han et al., 2024). Spearman correlation coefficients were calculated for all gene–metabolite pairs, and significantly correlated pairs with |r| > 0.8 and p < 0.05 were retained for visualization in four-quadrant plots. To identify common patterns between the two omics layers, we performed supervised integrative analysis using the two-way orthogonal partial least squares (O2PLS) model (version 1.0.0). The optimal number of joint and specific components was determined by 7-fold cross-validation, with model performance evaluated using R²X, R²Y, and Q² metrics. The global concordance between transcriptomic and metabolomic datasets was assessed by Procrustes analysis (n = 1,000 permutations, p < 0.05). For functional integration, differentially expressed genes (DEGs) and differentially accumulated metabolites (DAMs) were jointly mapped to KEGG pathways, and pathway enrichment was evaluated using a hypergeometric test with FDR < 0.05 as the significance threshold. To visualize the coordinated regulation of key metabolic pathways, the phenylpropanoid and flavonoid biosynthesis pathways were specifically extracted and displayed using integrated pathway maps, in which gene expression and metabolite accumulation patterns were overlaid with the same color-scaled gradient based on their normalized values across the five developmental stages.

### 2.7 Co-expression network construction

Weighted gene co-expression network analysis (WGCNA) was performed in R (version 4.2.0) using the WGCNA package (version 1.72-1). Only genes with FPKM > 1 in at least one sample were retained for analysis. To determine the optimal soft-thresholding power, we tested a range of powers from 1 to 30 and selected β = 9, which was the lowest power that achieved a scale-free topology fit index R² > 0.85. Gene co-expression modules were identified using the dynamic hybrid tree-cutting algorithm with a minimum module size of 50 and deepSplit = 2. Modules with highly similar expression profiles were merged at a merge cut height of 0.25. To identify modules significantly associated with floral development, module eigengenes were correlated with the developmental stage variables. For this analysis, the five developmental stages (T1–T5) were encoded as ordered numerical variables (1, 2, 3, 4, 5) according to their sequential progression. The resulting associations are referred to as module–stage associations, reflecting the correlation between co-expression modules and developmental stage progression rather than measured physiological traits. Within the modules of interest, genes with kME (module membership) > 0.3 were retained for further analysis. To prioritize hub candidate genes, we further selected the top 200 genes by intramodular connectivity (kIN) within each key module, and only connections with edge weights (absolute correlation coefficients) > 0.5 were retained for network visualization and downstream analysis. Hub candidate genes were defined as those satisfying the above criteria. It should be noted that these genes were identified based on correlation analyses and require further experimental validation. Functional enrichment analyses (GO and KEGG) were performed on the candidate hub genes using the same significance criteria (p < 0.05).

### 2.8 Validation by quantitative real-time PCR

To validate RNA-seq results, qRT-PCR was performed described (Pfaffl, 2001). Total RNA was reverse-transcribed into cDNA using the PrimeScript RT Reagent Kit (Takara, Dalian, China). Gene-specific primers were designed using Primer Premier 5.0. qRT-PCR amplification was conducted on a Bio-Rad CFX96 system using SYBR Green chemistry. The Actin gene was used as the internal reference. Relative gene expression levels were calculated using the 2^-^^ΔΔ^Ct method. Pearson correlation analysis was used to assess the consistency between RNA-seq and qRT-PCR data. Primer sequences, annealing temperatures, and amplicon sizes are listed in **Supplementary Appendix Table 2**.

## 3 Results

### 3.1 Dynamic transcriptome analysis during different developmental stages of sesame flowers

**Figure 1:**
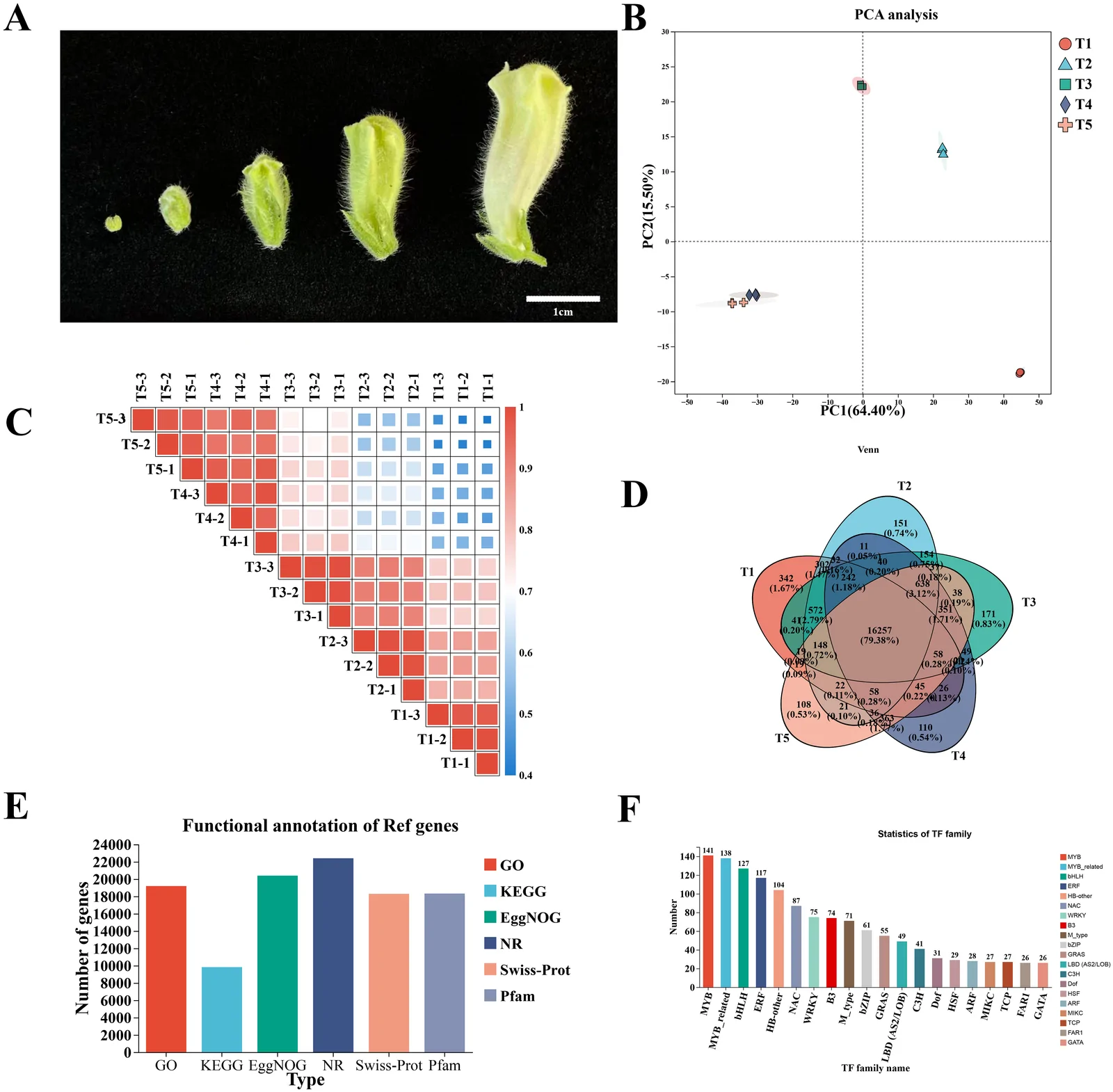
Results of transcriptome analysis of sesame flowers at five developmental stages. **(A)** Phenotypic observation of sesame flowers at five developmental stages (T1, T2, T3, T4, T5). Scale bar = 0.5cm. **(B)** Principal component analysis (PCA) plot based on all 15 samples (n = 3 biological replicates per stage). **(C)** Pearson correlation heatmap among all 15 samples based on normalized FPKM values; color gradient from blue (low correlation) to red (high correlation) indicates correlation coefficients. **(D)** Venn diagram showing the number of expressed genes (FPKM > 1 in at least one sample) shared and unique among the five developmental stages. **(E)** Bar chart of functional annotation of reference genes based on NR, Swiss-Prot, KEGG, and GO databases via EggNOG-mapper. **(F)** Bar chart of transcription factor (TF) family statistics classified by plant TF database (PlantTFDB).

To systematically decipher the dynamic regulatory networks governing gene expression during sesame floral development, we performed RNA-seq across five key developmental stages (T1–T5) spanning from young buds to mature flowers (**Figure 1A**). To ensure the reliability of transcriptomic analyses, we assessed RNA-sequencing data quality across all 15 samples. As summarized in **Supplementary Table 3**, each sample yielded 36.49–43.48 million clean reads, with overall mapping rates of 52.88%–75.02% and unique mapping rates of 52.08%–73.15%. High Q30 scores (95.99%–96.26%) and low error rates (~1.18%) confirmed the high quality and reliability of the RNA-seq data for downstream analyses. Principal component analysis (PCA) of the 15 RNA-seq samples revealed a clear developmental trajectory along PC1, which explained 64.40% of the variance (cumulative 79.90% with PC2), with samples sequentially ordered from T5 to T1. The 95% confidence ellipses of T1–T3 were well separated, indicating substantial transcriptomic reprogramming during early-to-mid stages, whereas T4 and T5 largely overlapped, suggesting transcriptional stabilization upon floral maturation (**Figure 1B**). High consistency among biological replicates (Pearson correlation coefficients > 0.9) and significantly reduced correlations between early (T1–T2) and late (T4–T5) stages further confirmed the excellent reproducibility and stage-specific dynamic nature of the RNA-seq data (**Figure 1C**). Venn diagram analysis identified a core transcriptome comprising 16,257 genes, representing 79.38% of all expressed genes, which were stably expressed across all five developmental stages (**Figure 1D**). Additionally, each stage contained a unique set of stage-specific genes, with 342 genes specific to T1, 151 to T2, 171 to T3, 110 to T4, and 108 to T5, implying distinct molecular processes unique to each developmental window. Functional annotation revealed that the vast majority of genes were successfully annotated in major public databases, including NR, Swiss-Prot, KOG, and KEGG (**Figure 1E**; **Supplementary Table 4**), providing a reliable foundation for subsequent analyses. Notably, we identified 1,334 transcription factors distributed among 20 major families, with ERF (117), bHLH (127), and MYB/MYB-related (279) family members being the most abundant, underscoring the central role of transcriptional regulation in orchestrating the complex regulatory network during sesame floral development (**Figure 1F**; **Supplementary Table 5**).

### 3.2 Temporal patterns and functional enrichment analysis of differentially expressed genes

**Figure 2:**
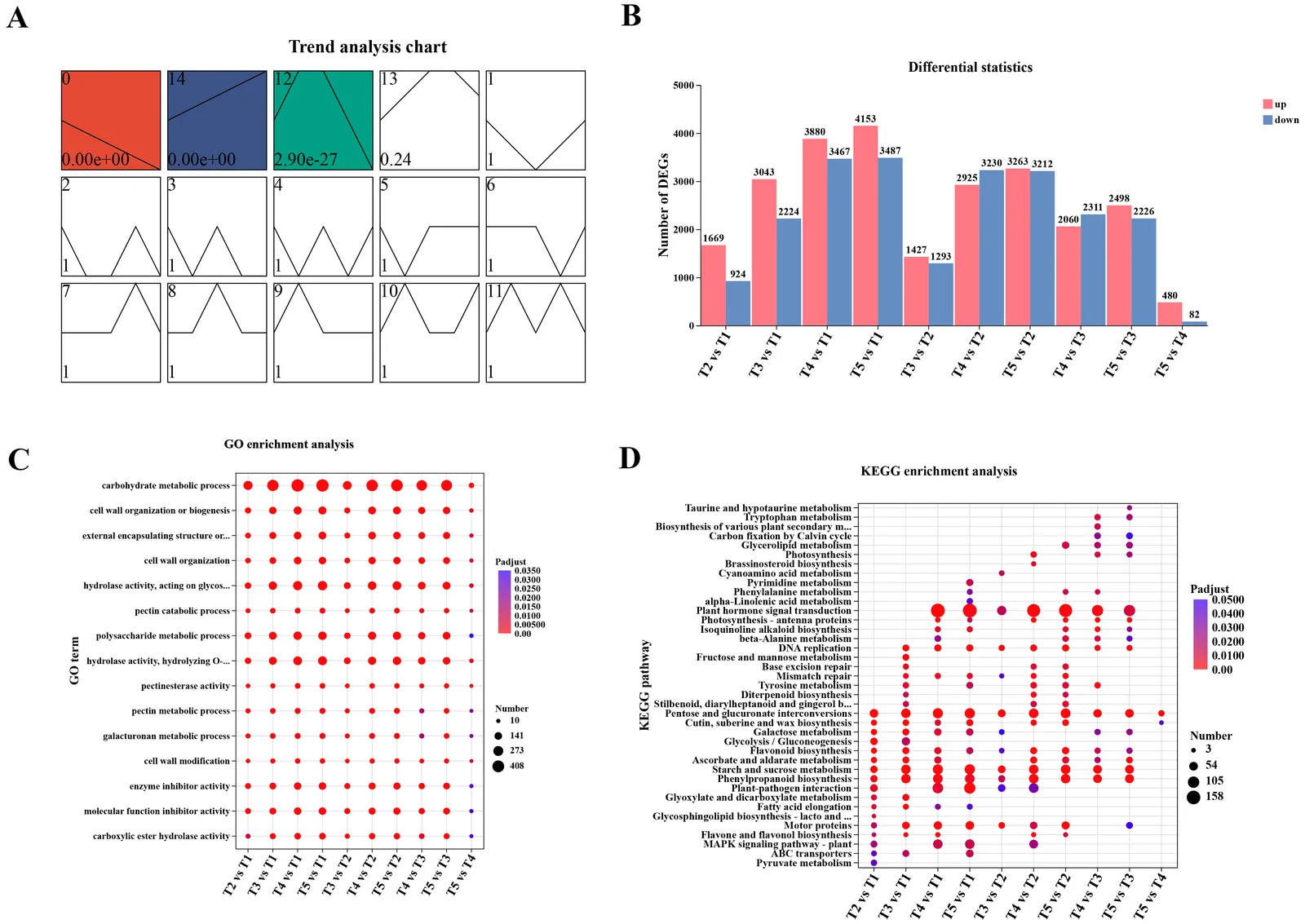
Results of transcriptome differential expression and functional enrichment analysis of sesame flowers at five developmental stages. **(A)** Trend analysis of gene expression patterns; only profiles with FDR < 0.05 were considered significant. **(B)** Bar chart of differentially expressed gene (DEG) statistics between adjacent stages. DEGs were identified using DESeq2 with thresholds of FDR < 0.05 and |log_2_ fold change| ≥ 1. **(C)** Dot plot of GO enrichment analysis for DEGs; the top 20 significantly enriched GO terms are shown (FDR < 0.05). The dot size represents the number of enriched genes, and the color gradient represents the FDR-adjusted p-value. **(D)** Dot plot of KEGG pathway enrichment analysis for DEGs; the top 20 significantly enriched pathways are shown (FDR < 0.05). The dot size represents the number of enriched genes, and the color gradient represents the FDR-adjusted p-value.

Temporal expression pattern analysis using Short Time-series Expression Miner (STEM) identified 15 significant expression profiles across the five developmental stages, capturing the full spectrum of gene expression dynamics during floral development (**Figure 2A**; **Supplementary Table 6**). Through pairwise comparisons between adjacent developmental stages, we systematically identified differentially expressed genes (DEGs) and characterized their temporal dynamics. Statistical analysis revealed that compared to the T1 stage, the total number of DEGs generally increased during development, with the proportion of up-regulated genes gradually rising, indicating progressive activation of development-specific genetic programs (**Figure 2B**). Specifically, the T4 vs T1 comparison showed the highest number of DEGs, with 4,153 up-regulated and 3,487 down-regulated genes, reflecting massive transcriptional reprogramming during early-to-mid development. In contrast, the T5 vs T4 comparison contained the fewest DEGs, totaling 562 genes with 480 up-regulated and 82 down-regulated, indicating that gene expression stabilizes upon entering the late developmental stage, consistent with the PCA results. GO functional enrichment analysis revealed that DEGs were significantly associated with oxidoreductase activity, carbohydrate metabolic process, and transporter activity, reflecting the diverse molecular events accompanying floral development (**Figure 2C**). KEGG pathway analysis further showed significant enrichment (P < 0.05) in plant hormone signal transduction, phenylalanine metabolism, and starch and sucrose metabolism, which contained numerous DEGs and were dynamically regulated across stages (**Figure 2D**). Energy metabolism pathways, including photosynthesis and carbon fixation, were also enriched, reflecting dynamic adjustments in material and energy metabolism that support cell proliferation, organ differentiation, and subsequent reproductive processes. These stage-specific DEGs and their associated pathways provide a foundation for identifying key regulatory modules governing floral development, which we further explored through weighted gene co-expression network analysis.

### 3.3 Metabolic profiling during sesame floral development

**Figure 3:**
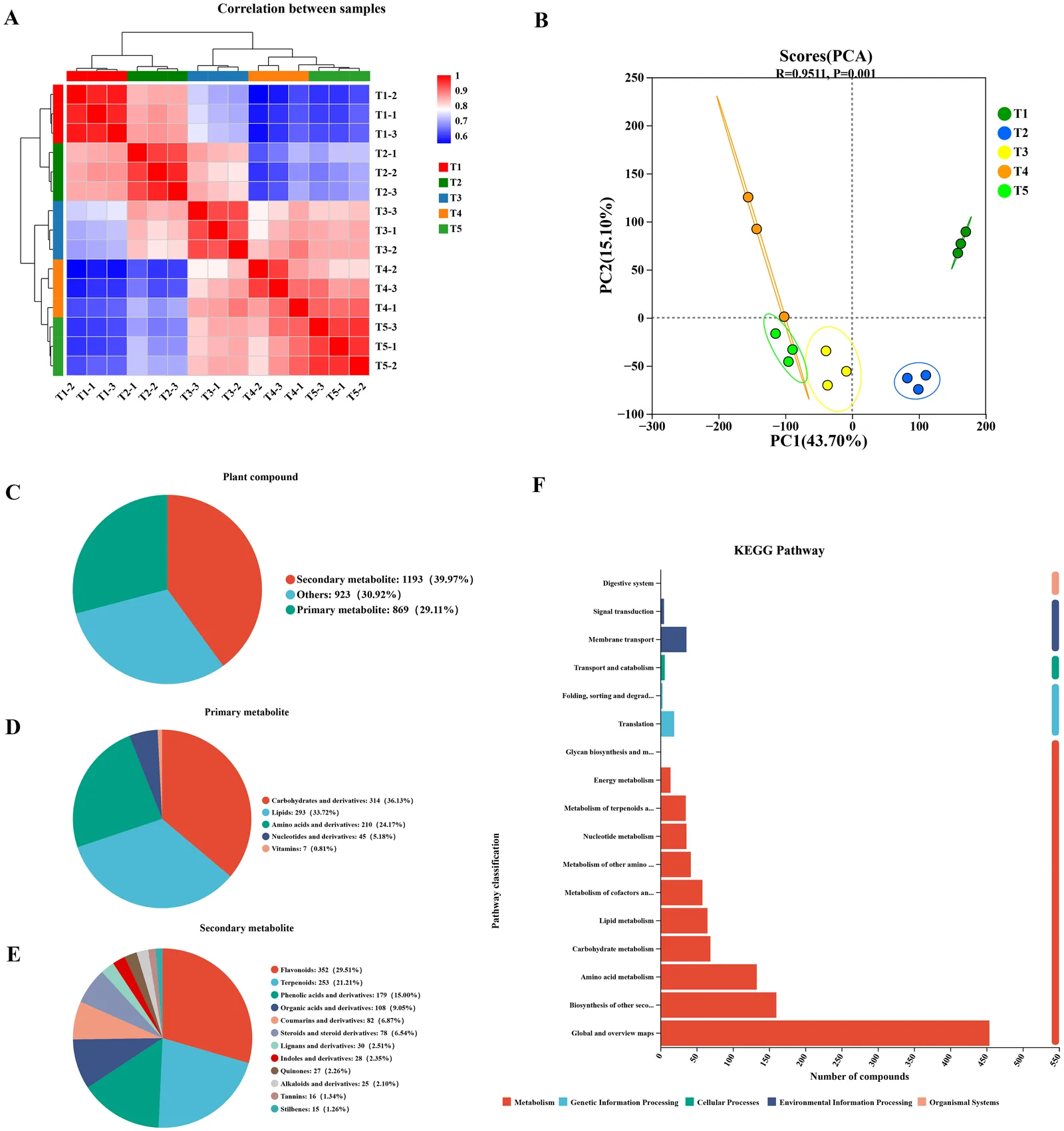
Metabolome analysis of sesame flowers at five developmental stages. **(A)** Pearson correlation heatmap among all 15 metabolomic samples (n = 3 biological replicates per stage) based on normalized peak intensity data; color gradient from blue (low correlation) to red (high correlation) indicates correlation coefficients. **(B)** Principal component analysis (PCA) score plot based on all 15 samples (n = 3 biological replicates per stage). **(C)** Plant compound classification composition showing the number and percentage of metabolites in each class identified via HMDB and Metlin databases. **(D)** Primary metabolite classification with compound counts and percentages. **(E)** Secondary metabolite classification with compound counts and percentages. **(F)** KEGG pathway classification distribution showing the number of metabolites assigned to each KEGG pathway category.

Metabolomes across the five developmental stages were characterized using untargeted liquid chromatography coupled with tandem mass spectrometry (LC-MS/MS) to capture the full spectrum of small-molecule metabolites and their dynamic changes during floral development. It should be noted that this approach provides relative quantification based on normalized peak intensities rather than absolute concentrations (μg/g FW). All metabolite abundance values reported in this study reflect relative abundance rather than absolute content. PCA of the metabolomic data robustly distinguished all five developmental stages, exhibiting a highly congruent developmental trajectory with the transcriptome dataset (r = 0.951, P < 0.001), affirming the reliability and biological coherence of both multi-omics datasets (**Figures 3A, B**). Furthermore, we identified a total of 2,985 metabolites, providing a comprehensive metabolic landscape of sesame floral development. Notably, secondary metabolites, comprising 1,193 compounds (39.97%), exceeded primary metabolites in number, highlighting the prominence and functional importance of specialized metabolic pathways during floral development. Detailed classification revealed that flavonoids formed the most abundant group within secondary metabolites, while carbohydrates, lipids, and amino acids were the major primary metabolite classes, reflecting the central roles of both primary energy supply and secondary metabolite biosynthesis in floral organ development (**Figures 3C–E**; **Supplementary Table 7**). KEGG pathway annotation showed that metabolism-related pathways contained the highest number of metabolites, with the most abundant being Global and overview maps (454 metabolites), Biosynthesis of secondary metabolites (160 metabolites), and Amino acid metabolism (133 metabolites), indicating that extensive activation of diverse metabolic pathways during sesame floral development forms a critical material foundation for cellular proliferation, organ morphogenesis, and subsequent reproductive competence (**Figure 3F**).

### 3.4 Differential accumulated metabolite analysis

**Figure 4:**
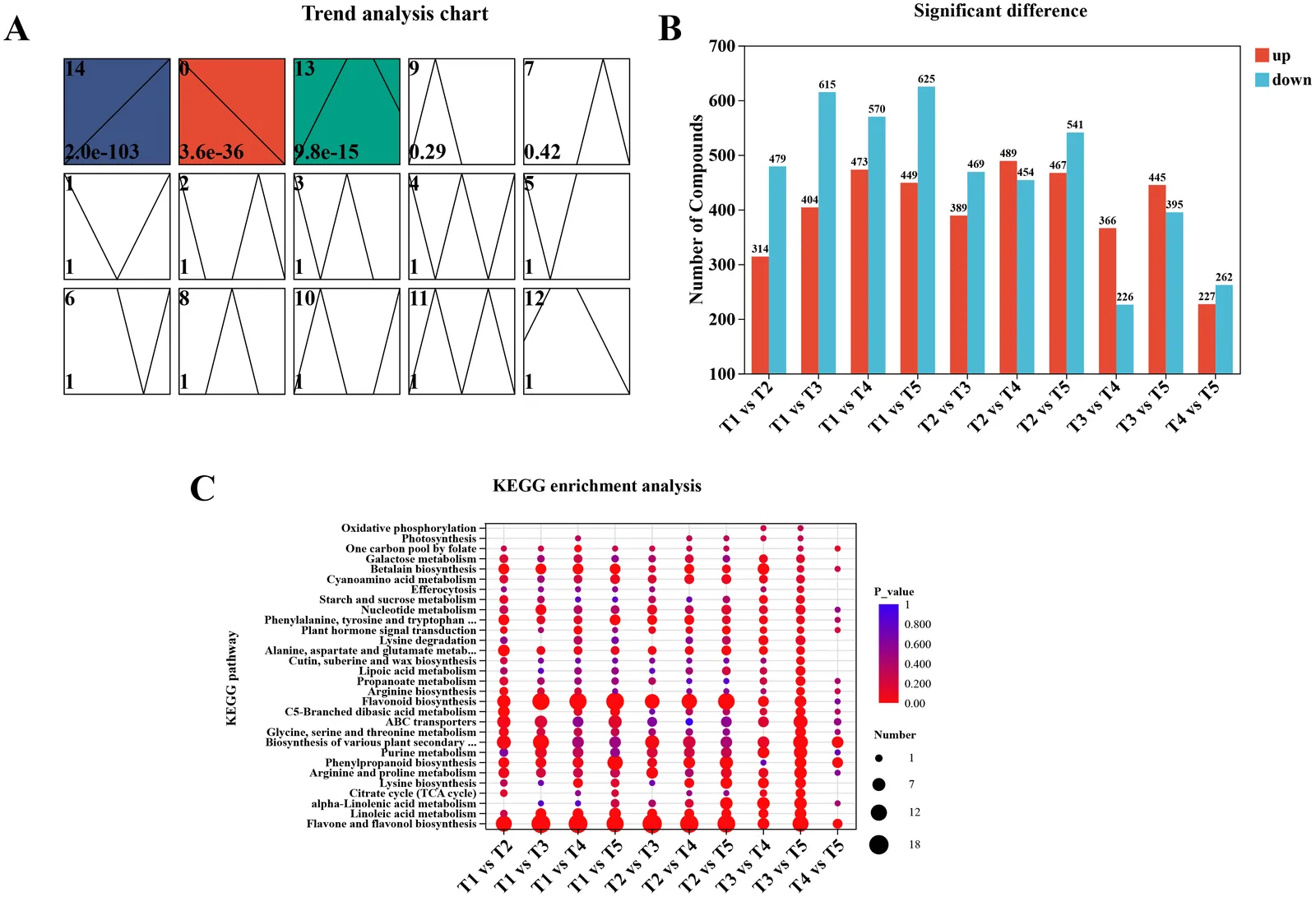
Temporal dynamics of metabolite accumulation and differential metabolite enrichment in sesame flowers at five developmental stages. **(A)** Trend analysis of metabolite expression patterns; only profiles with FDR < 0.05 were considered significant. **(B)** Statistical bar chart of significantly differential metabolites between adjacent stages. DAMs were identified with thresholds of VIP > 1 and p < 0.05 (Student’s t-test). **(C)** Dot plot of KEGG enrichment analysis for DAMs across all comparison groups. The vertical axis represents significantly enriched KEGG pathways, the horizontal axis represents different sample comparison groups. Dot color represents the p-value (color gradient from red for low p-value to blue for high p-value), and dot size represents the number of enriched metabolites.

Time-series clustering of metabolite abundance identified 15 distinct clusters with characteristic accumulation patterns across developmental stages (**Figure 4A**; **Supplementary Table 8**). Among these, Clusters 14, 0, and 13 showed extremely high statistical significance (P-values = 2.0e-103, 3.6e-36, and 9.8e-15, respectively): Cluster 14 contained 237 metabolites exhibiting continuous up-accumulation throughout development; Cluster 13 contained 95 metabolites significantly up-accumulated specifically during T3 and T4; while Cluster 0 contained 135 metabolites showing continuous down-accumulation, systematically uncovering the stage-specific accumulation patterns of metabolites during sesame floral development. Differential accumulated metabolite (DAM) analysis revealed that the T1 vs T5 comparison contained the highest number of DAMs, with 625 up-accumulated and 449 down-accumulated metabolites, reflecting profound metabolic reprogramming from early bud to mature flower. In contrast, the T4 vs T5 comparison showed the fewest DAMs, totaling 489 metabolites with 227 up-accumulated and 262 down-accumulated, indicating that metabolite composition stabilizes during late developmental stages, consistent with the reduced transcriptomic variation observed at these stages (**Figure 4B**).

KEGG enrichment analysis mapped these DAMs to the top 30 abundant metabolic pathways, including Flavonoid biosynthesis, Flavone and flavonol biosynthesis, Linoleic acid metabolism, Betalain biosynthesis, and Alanine, aspartate and glutamate metabolism (P-value < 0.05) (**Figure 4C**). Among these, Flavonoid biosynthesis, Flavone and flavonol biosynthesis, and Phenylpropanoid biosynthesis pathways contained substantial numbers of differentially accumulated metabolites, showing high consistency with the transcriptome enrichment results. This strong convergence between transcriptomic and metabolomic data provides robust pathway-level evidence for understanding the material basis and dynamic metabolic regulatory mechanisms governing sesame floral development.

### 3.5 Integrated analysis of transcriptome and metabolome across five developmental stages of sesame flower

**Figure 5:**
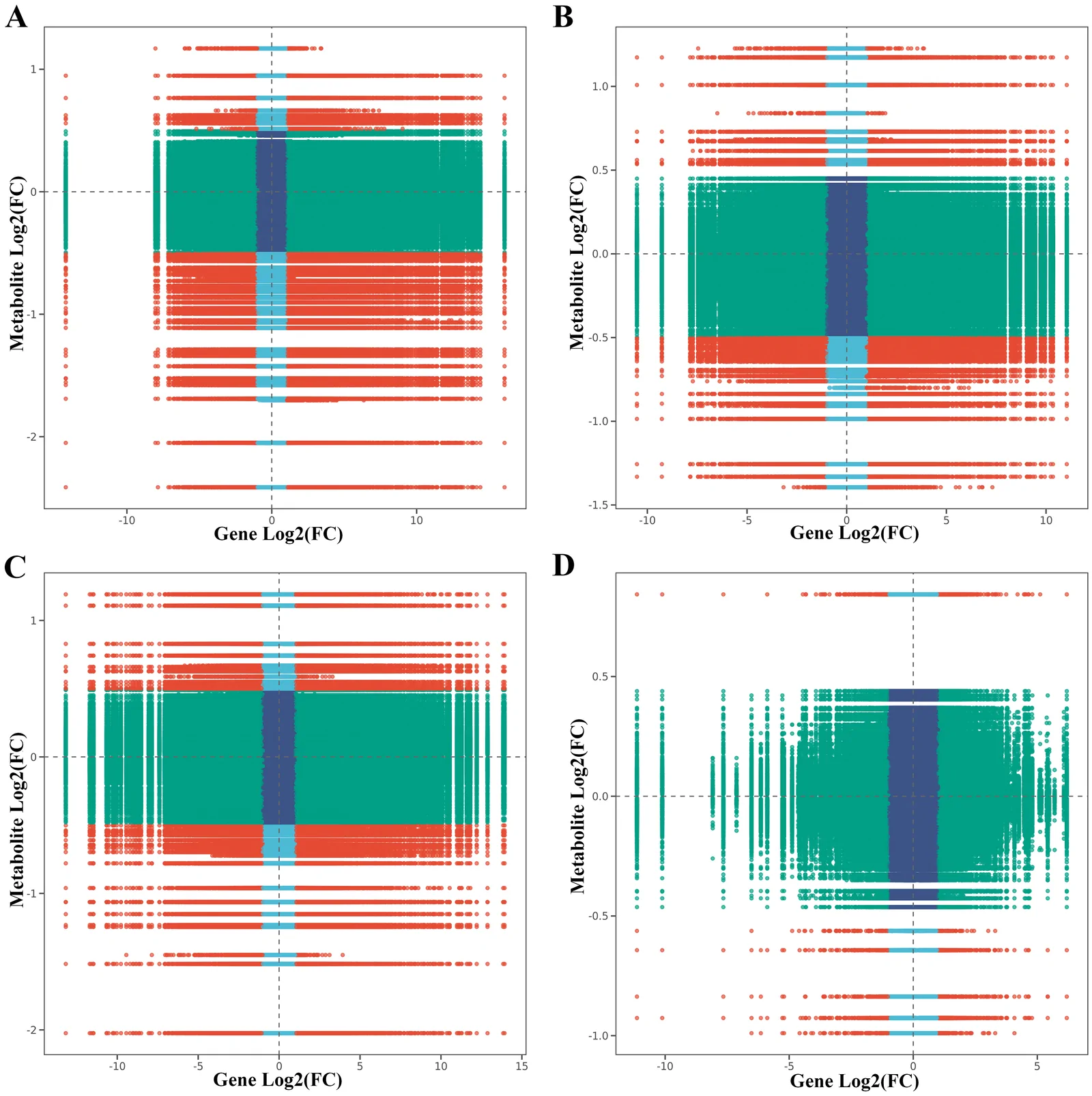
Four-quadrant differential association plots of differential metabolites (DAMs) and differentially expressed genes (DEGs) across adjacent developmental stages in sesame flowers. **(A–D)** Correlation analysis between the transcriptome and metabolome during the transitions of **(A)** T1 vs. T2, **(B)** T2 vs. T3, **(C)** T3 vs. T4, and **(D)** T4 vs. T5. DEGs were identified with thresholds of FDR < 0.05 and |log_2_ fold change| ≥ 1; DAMs were identified with thresholds of VIP > 1 and p < 0.05. Each dot represents a gene–metabolite pair with significant Spearman correlation (|r| > 0.8, p < 0.05). Color coding of scatter points: red dots represent both DAMs and DEGs; light blue dots indicate DAMs only with non-DEGs; green dots indicate DEGs only with non-DAMs; and dark blue dots represent both non-significant metabolites and genes.

To systematically characterize the coordinated regulatory relationships between transcripts and metabolites across adjacent developmental stages, we constructed four-quadrant differential association scatter plots by integrating DEGs and DAMs (**Figures 5A–D**). Across all four transitions, gene expression changes consistently exhibited broader dynamic ranges than metabolite changes, indicating that transcriptional reprogramming serves as the primary driver of stage-specific metabolic shifts during sesame floral development. During the early transition from T1 to T2, gene expression changes were most pronounced (Log_2_FC range: approximately -10 to 15), while metabolite changes remained relatively concentrated (-2 to 1) (**Figure 5A**). From T2 to T3, the range of gene Log_2_FC values narrowed slightly, whereas metabolite variation expanded modestly, suggesting a progressive coordination between transcriptional and metabolic responses during mid-development (**Figure 5B**). Notably, at the critical T3-to-T4 transition—corresponding to petal expansion to stamen maturation—transcriptional reprogramming intensity reached its peak, with extreme Log_2_FC values exceeding 15 and falling below -10, while metabolite changes remained relatively stable (-2 to 1) (**Figure 5C**). In contrast, by the late T4-to-T5 transition, both transcriptional and metabolic changes were markedly attenuated, and the proportion of concordant gene-metabolite trends dropped to its lowest level among all comparisons, reflecting the stabilization of the transcriptomic and metabolic landscapes upon floral maturation (**Figure 5D**).

**Figure 6:**
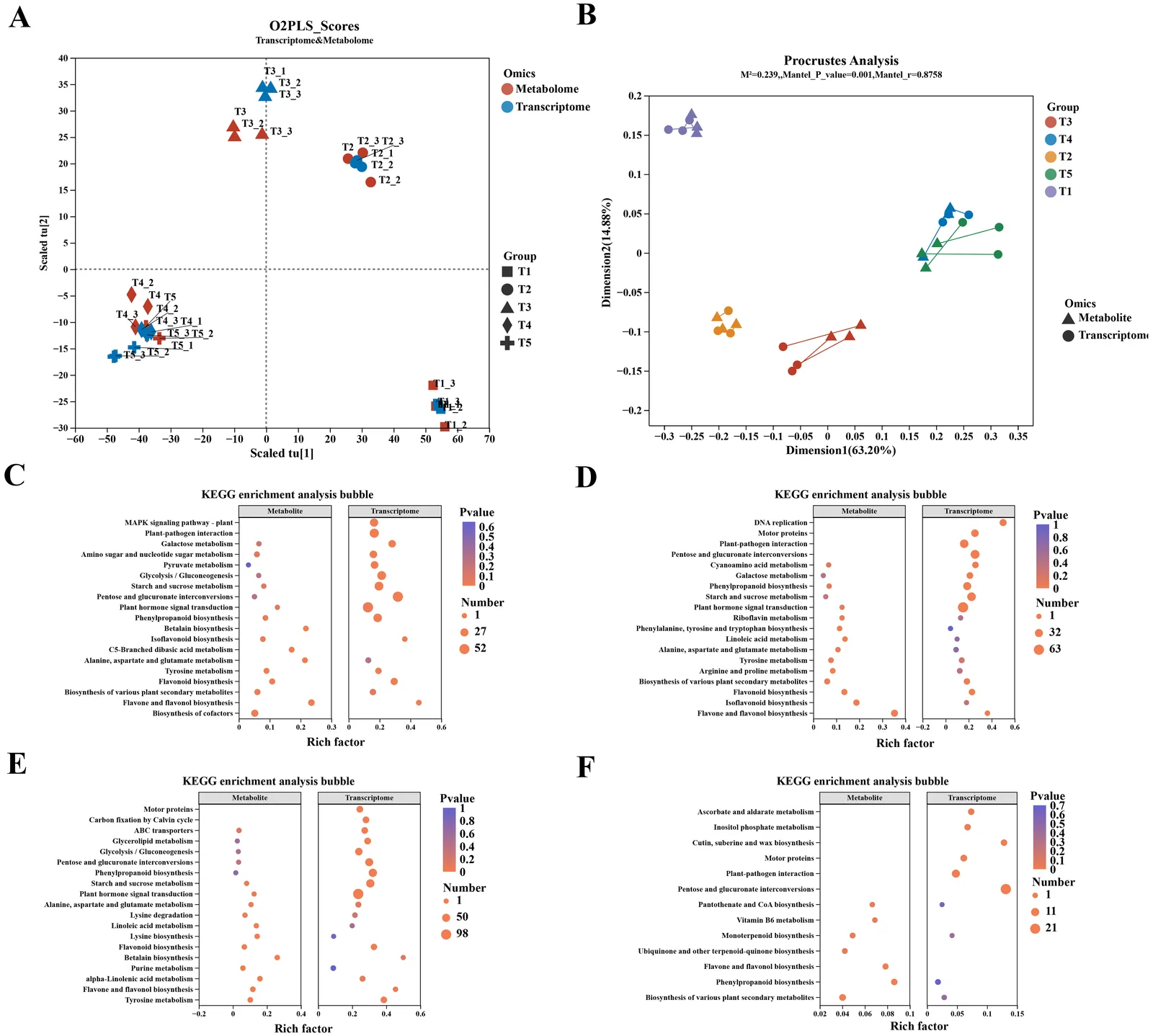
Integrated multi-omics analysis of transcriptome and metabolome during sesame floral development. **(A)** O2PLS score plot: Distribution of transcriptome (blue) and metabolome (red) datasets across developmental stages T1 to T5. The optimal number of components was determined by 7-fold cross-validation; model performance was evaluated using R²X, R²Y, and Q² metrics. **(B)** Procrustes analysis: Assessment of the association between metabolome (triangles) and transcriptome (circles) datasets based on 1,000 permutations; p < 0.05 indicates significant concordance. **(C–F)** KEGG enrichment analysis between adjacent stages: Co-enrichment analysis of DEGs and DAMs for transitions **(C)** T1 vs. T2, **(D)** T2 vs. T3, **(E)** T3 vs. T4, and **(F)** T4 vs. T5 with FDR < 0.05 as the significance threshold for enriched pathways.

To further assess global concordance between the two omics layers, we performed O2PLS and Procrustes analyses (**Figures 6A, B**). The O2PLS score plot revealed strong stage-specific clustering for both transcriptomic (blue) and metabolomic (red) datasets, with T4 and T5 samples positioned in close proximity, consistent with the PCA results (**Figure 6A**). Procrustes analysis further validated the overall concordance between the two datasets, as samples from the same developmental stage (metabolomic as triangles, transcriptome as circles) were closely aligned in the two-dimensional space (**Figure 6B**). These results demonstrate a high degree of global coordination between transcriptional and metabolic reprogramming throughout sesame floral development. KEGG co-enrichment analysis of DEGs and DAMs across adjacent stages revealed a dynamic landscape of pathway activation (**Figures 6C–F**). During early development (T1-T2 and T2-T3), co-enriched pathways were predominantly associated with primary metabolism and stress responses, including plant hormone signal transduction, pentose and glucuronate interconversions, glycolysis/gluconeogenesis, starch and sucrose metabolism, and plant-pathogen interaction, indicating that early floral development is characterized by active energy metabolism and hormone-mediated regulatory programs. From T3 to T4, the number of co-enriched pathways increased to 18, with phenylpropanoid and flavonoid biosynthesis pathways becoming prominently co-enriched alongside the aforementioned primary metabolic pathways. This expansion of co-enriched pathways at the T3-T4 transition indicates that mid-to-late floral development involves simultaneous activation of both primary metabolic support and secondary metabolic specialization. By the late transition (T4-T5), the number of co-enriched pathways sharply decreased to only four, with DAMs primarily enriched in secondary metabolite pathways including phenylpropanoid and flavonoid biosynthesis, while DEGs were mainly enriched in pentose and glucuronate interconversions and plant-pathogen interaction. This marked reduction in co-enriched pathways at the late stage further supports the notion that transcriptional and metabolic landscapes stabilize upon floral maturation, with secondary metabolism persisting as a major functional module. The progressive increase and subsequent decline in the number of co-enriched pathways across developmental stages highlight a tightly coordinated and stage-dependent multi-omics regulatory program underlying sesame floral development.

### 3.6 Co-expression network identification of core modules associated with floral development

**Figure 7:**
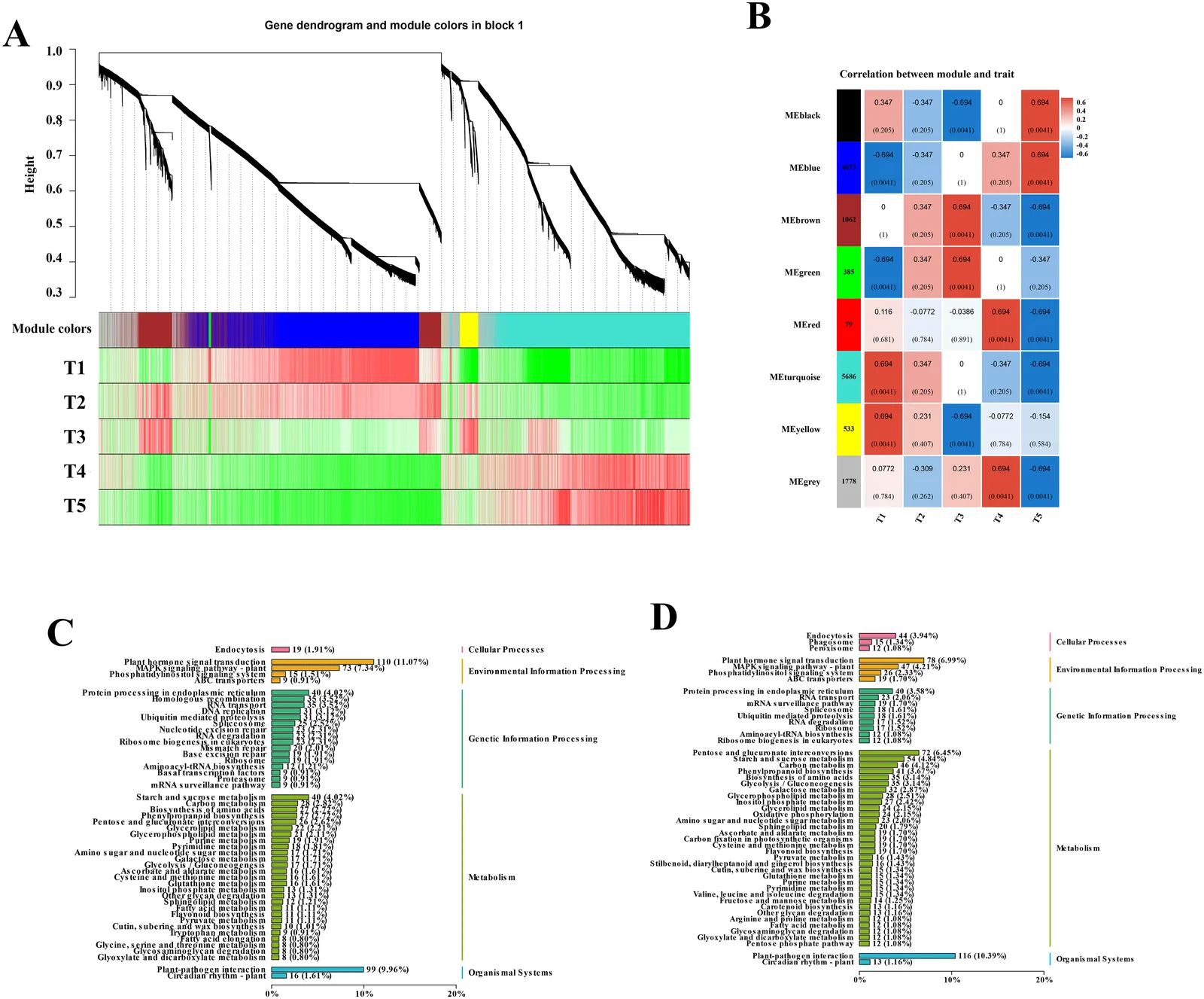
Results of weighted gene co-expression network analysis (WGCNA) of sesame flowers at five developmental stages. **(A)** Gene dendrogram and module colors in block 1 generated using the dynamic hybrid tree-cutting algorithm (minModuleSize = 50, deepSplit = 2, mergeCutHeight = 0.25); a soft-thresholding power of β = 24 was selected to achieve scale-free topology (R² > 0.85). **(B)** Correlation heatmap between modules and traits (T1–T5 developmental stages encoded as ordered numerical variables 1–5). Each cell represents the correlation coefficient between a module eigengene and a developmental stage; numbers in parentheses indicate p-values. **(C)** KEGG functional classification diagram of key genes in the MEblue module; genes with kME > 0.3 were retained for functional annotation; top enriched KEGG pathways are shown. **(D)** KEGG functional classification diagram of key genes in the MEturquoise module; genes with kME > 0.3 were retained for functional annotation; top enriched KEGG pathways are shown.

To identify co-regulated gene modules at the genome-wide level, we constructed a weighted gene co-expression network using the WGCNA algorithm. Hierarchical clustering partitioned genes into multiple co-expression modules (**Figure 7A**). Module–stage correlation analysis identified a blue module positively correlated with developmental progression and a turquoise module negatively correlated with stage progression (**Figure 7B**; **Supplementary Table 9**). The blue and turquoise modules exhibited moderate and high module significance (MS) values, respectively, and gene significance (GS) was strongly correlated with module membership (kME) in both modules (blue: cor = −0.694, p < 0.0041; turquoise: cor = 0.694, p < 0.0041; **Supplementary Figure 1**). These two modules therefore represent gene sets that are either progressively activated or gradually repressed during floral development. KEGG enrichment analysis revealed distinct functional specializations between the two modules. Genes in the blue module (positively correlated with development) were primarily enriched in metabolic pathways, particularly pentose and glucuronate interconversions (7 genes, 11.86%) and phenylpropanoid biosynthesis (5 genes, 8.46%), as well as plant hormone signal transduction (4 genes, 6.78%) (**Figure 7C**), suggesting that this module coordinates secondary metabolism and hormone-mediated processes during floral maturation. In contrast, the turquoise module (negatively correlated with development) was predominantly enriched in genetic information processing pathways, including DNA replication, base excision repair, nucleotide excision repair, ubiquitin-mediated proteolysis, homologous recombination, and mismatch repair, together with plant hormone signal transduction (10 genes, 15.87%), purine/pyrimidine metabolism, and starch and sucrose metabolism (**Figure 7D**). This functional composition indicates that the turquoise module is primarily associated with active cell proliferation, DNA maintenance, and energy supply during early floral development.

**Figure 8:**
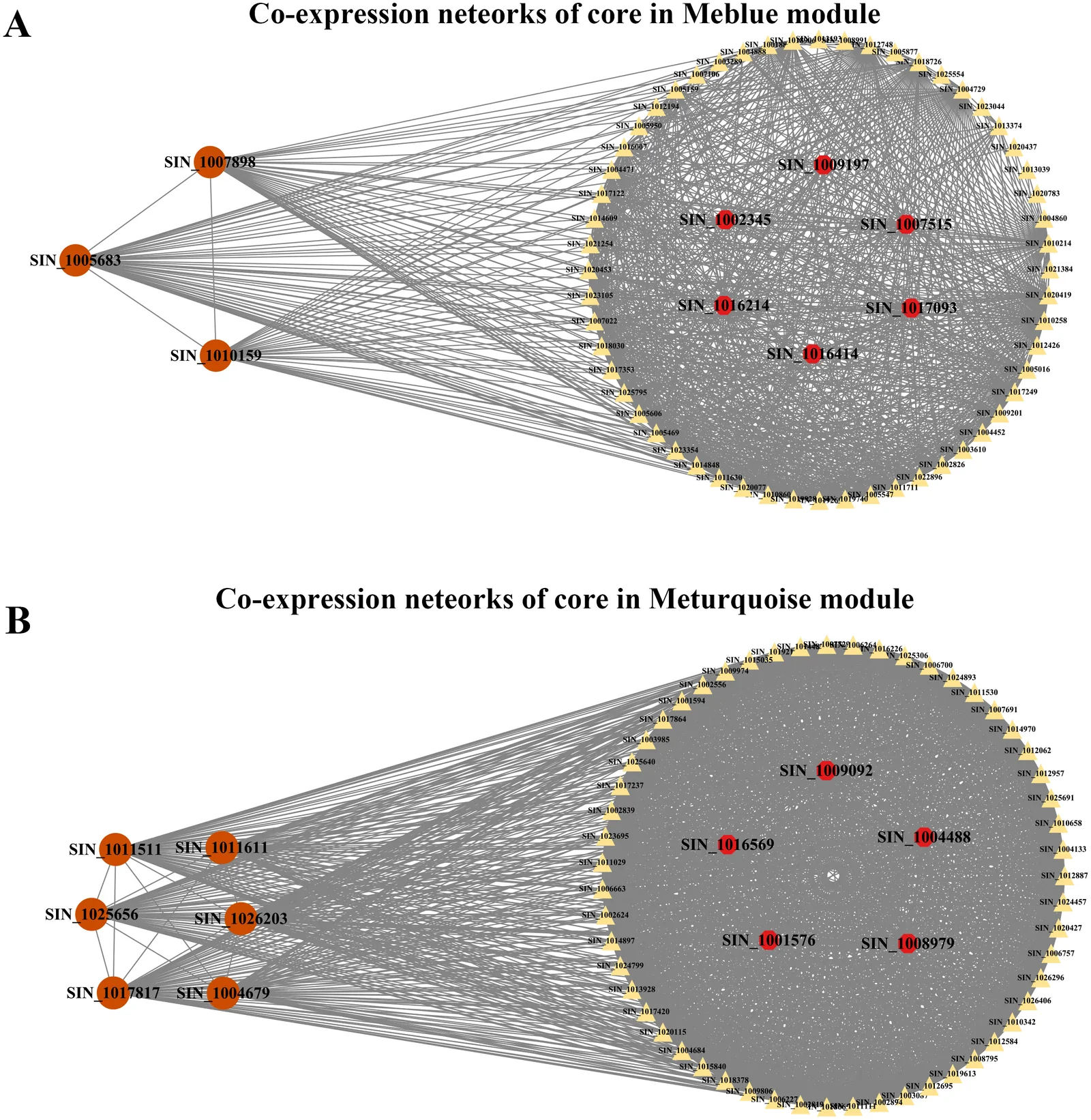
Co-expression networks in key modules. **(A)** Co-expression network of the MEblue module; only connections with edge weights (absolute correlation coefficients) > 0.5 are displayed; nodes represent genes, node size indicates intramodular connectivity (kIN), and edge thickness indicates correlation strength. **(B)** Co-expression network of the MEturquoise module; only connections with edge weights (absolute correlation coefficients) > 0.5 are displayed; nodes represent genes, node size indicates intramodular connectivity (kIN), and edge thickness indicates correlation strength.

To evaluate the topological importance of genes within the two key modules, we assessed intramodular connectivity (kIN) and module membership (kME) for all genes in the blue and turquoise modules (**Supplementary Table 10**). In the blue module, all 74 hub genes exhibited high connectivity, with kIN values ranging from 1568.73 to 1665.37 and kME values ranging from 0.9628 to 0.9961. Visualization of the co-expression network within the blue module revealed that these highly connected genes are centred on transcription factors including MYB (SIN_1007898, SIN_1010159) and BAG (SIN_1005683), alongside structural genes such as pectinesterase (SIN_1016214, SIN_1009197), pollen receptor-like kinase (SIN_1007515), auxin transporter protein (SIN_1017093), gibberellin-regulated protein (SIN_1016414), and flavanone 3-dioxygenase (SIN_1002345/F3H) (**Figure 8A**). The dense interconnections among these genes suggest that MYB transcription factors may regulate structural genes to coordinate secondary metabolism and cell wall modification during floral development. In the turquoise module, the 64 hub genes displayed even higher connectivity, with kIN values ranging from 1987.76 to 2074.27 and kME values from 0.9809 to 0.9981. SIN_1014970 exhibited the highest connectivity (kIN = 2074.27, kME = 0.9981), followed by SIN_1002894 (kIN = 2059.04, kME = 0.9970) and SIN_1015035 (kIN = 2059.77, kME = 0.9958). The co-expression network of the turquoise module comprised multiple transcription factors including OFP (SIN_1011611), ZF-HD (SIN_1011511), AP2/ERF (SIN_1025656, SIN_1026203), GRAS (SIN_1017817), and MYB (SIN_1004679), as well as functional genes including EMS1 (SIN_1016569), MCM (SIN_1001576), auxin efflux carrier component (SIN_1008979), auxin transporter-like protein (SIN_1004488), and serine/threonine protein kinase (SIN_1009092) (**Figure 8B**). This network architecture implies that transcription factors in the turquoise module may exert regulatory functions in early-stage growth and differentiation through hormone signal transduction pathways. The high kME and kIN values across both modules indicate that these genes are not only strongly representative of their respective modules but also occupy central positions within the co-expression networks. Collectively, the WGCNA results reveal two functionally contrasting co-expression modules that respectively support early proliferation and late maturation processes during sesame floral development, and provide a set of candidate hub genes for future functional studies.

### 3.7 Integrative analysis of transcriptome and metabolome

**Figure 9:**
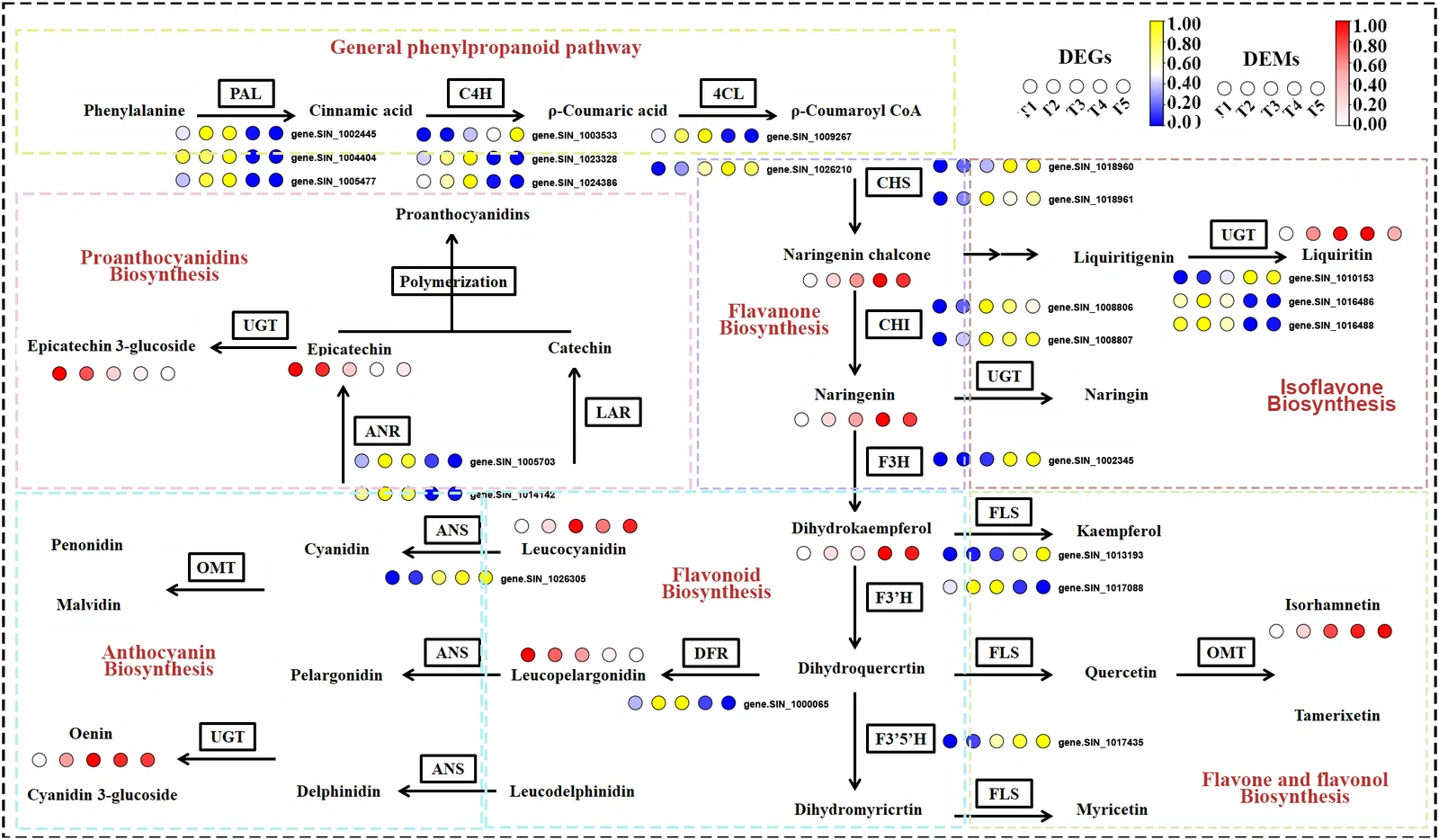
Expression profiling of genes and metabolites in the flavonoid biosynthesis pathway during sesame floral development. Differentially expressed genes (DEGs) are represented by colored circles with color gradients corresponding to normalized expression levels (log_2_-transformed FPKM) at stages T1–T5; differentially accumulated metabolites (DAMs) are represented by colored circles with color gradients corresponding to relative accumulation levels (normalized peak intensities) at stages T1–T5. Red indicates high expression/accumulation and blue indicates low expression/accumulation. All DEGs and DAMs shown were identified with FDR < 0.05, |log_2_ fold change| ≥ 1, VIP > 1, and p < 0.05 as applicable.

Given that both transcriptome and metabolome data highlighted the importance of phenylpropanoid/flavonoid metabolic pathways, we conducted an integrated analysis of this pathway to characterize the coordinated expression patterns of key enzyme genes and corresponding metabolite accumulation across developmental stages (**Figure 9**; **Supplementary Tables 11**, **12**). In the biosynthetic pathway from phenylalanine to various flavonoid products, 12 key enzyme genes-including phenylalanine ammonia-lyase (*PAL*), cinnamate 4-hydroxylase (*C4H*), 4-coumarate:CoA ligase (*4CL*), chalcone synthase (*CHS*), chalcone isomerase (*CHI*), flavanone 3-hydroxylase (*F3H*), flavonol synthase (*FLS*), flavonoid 3′,5′-hydroxylase (*F3′5′H*), UDP-glycosyltransferase (*UGT*), anthocyanidin synthase (*ANS*), dihydroflavonol 4-reductase (*DFR*), and anthocyanidin reductase (*ANR*) *-*exhibited differential expression patterns, while 10 related metabolites—including naringenin chalcone, naringenin, dihydrokaempferol, liquiritin, isorhamnetin, leucopelargonidin, oenin, epicatechin, and epicatechin 3-glucoside—were annotated within this pathway. Specifically, in the flavanone synthesis branch, *CHS*, *CHI*, and *F3H* genes (SIN_1018960, SIN_1008807, SIN_1002345) and metabolites such as naringenin chalcone and naringenin were significantly up-regulated during T4 and T5, indicating active flavanone synthesis in late floral development. In the flavonoid biosynthesis branch, *DFR* showed high expression during early stages, consistent with the accumulation trend of leucopelargonidin. In the proanthocyanidin branch, *ANR* expression aligned with epicatechin accumulation dynamics. For glycoside synthesis, *UGT* genes SIN_1010153 (potentially associated with liquiritin and oenin) were highly expressed at T4–T5, while SIN_1016486 and SIN_1016488 (potentially related to epicatechin 3-glucoside) were significantly up-regulated during early-to-mid stages. Notably, the sequential activation of these pathway branches followed a clear temporal order: upstream precursor synthesis genes peaked at T3, whereas downstream branch-specific genes reached their maxima at T4–T5, corresponding to the active accumulation of flavonoid end-products during late floral maturation (**Figure 9**). Collectively, gene expression and metabolite accumulation in the phenylpropanoid-flavonoid pathway exhibited stage specificity, with regulatory peaks sequentially forming at different developmental periods (T1–T2 for early precursor synthesis, T3 for intermediate steps, and T4–T5 for downstream branch metabolism), collectively constituting a dynamic regulatory network of secondary metabolism during sesame floral development.

### 3.8 Real-time quantitative PCR analysis of 10 genes across five developmental stages of sesame flower

**Figure 10:**
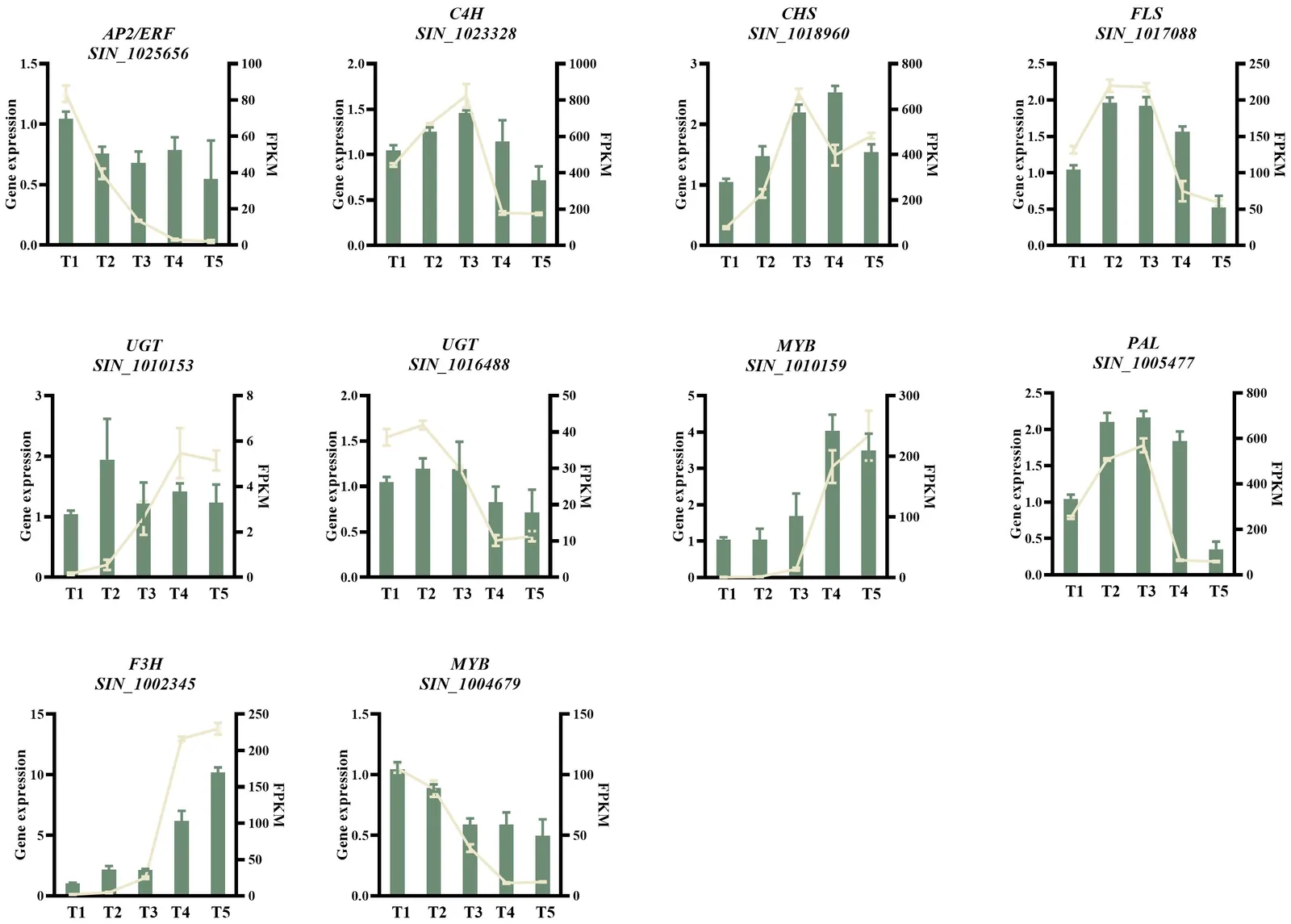
qRT-PCR validation of ten candidate genes during sesame floral development. The green bars represent the relative expression levels determined by qRT-PCR (left Y-axis; values represent mean fold change relative to T1 stage, normalized to the internal reference gene; error bars indicate standard deviation (SD) of three biological replicates), and the pale yellow lines indicate the expression trends derived from transcriptome (RNA-Seq) data (right Y-axis; normalized FPKM values). Statistical significance was determined by Student’s t-test (p < 0.05, p < 0.01, p < 0.001). Error bars represent the standard deviation (SD) of three biological replicates for qRT-PCR data.

To validate the RNA-seq data, we examined the temporal expression profiles of ten flavonoid pathway genes—including structural genes (*C4H*, *CHS*, *FLS*, *F3H*, *PAL*, *UGT*) and transcription factors (*AP2/ERF*, *MYB*)—across five developmental stages (T1–T5) by qRT-PCR (**Figure 10**). The qRT-PCR results (gray bars) and RNA-seq FPKM values (line graphs) were generally well matched, and three distinct expression patterns emerged among the candidate genes. The first group, comprising *C4H*, *CHS*, *FLS*, *PAL*, and *UGT* (SIN_1016488), showed a clear rise-and-fall pattern: transcript levels gradually increased from T1, peaked at T2–T3, and declined sharply at T4–T5. Given that these genes encode core rate-limiting enzymes in the phenylpropanoid and flavonol pathways, their mid-stage upregulation points to T2–T3 as a critical window for active flavonoid biosynthesis in sesame flowers. The second group, including *AP2/ERF*, *MYB* (SIN_1004679), and *UGT* (SIN_1010153), exhibited maximum expression at T1 with steady downregulation thereafter, suggesting that these transcriptional regulators may act to prevent premature activation of flavonoid biosynthesis at early flowering stages. The third group, consisting of *F3H* and *MYB* (SIN_1010159), displayed low expression from T1 to T3, then rose sharply at T4 and reached the highest level at T5, implicating their roles in sustaining flavonoid modification and accumulation during late floral maturation. Overall, the qRT-PCR expression trends were largely consistent with the RNA-seq FPKM profiles across all ten genes. The timing of upregulation, peak expression, and downregulation coincided well between the two platforms, with only minor discrepancies in amplitude at certain stages and no conflicting directional changes. These results confirm the reliability of our transcriptomic data and validate the authentic expression dynamics of key flavonoid pathway genes during sesame flower development.

## 4 Discussion

Floral development is a precisely regulated, hierarchically complex biological process that determines plant reproductive success and directly influences crop yield. As an economically and nutritionally important oilseed crop, sesame (Sesamum indicum L.) exhibits unique floral traits shaped by its indeterminate inflorescence and short-day flowering habit. Although floral regulatory mechanisms have been well characterized in model plants and major oilseed crops, the stage-specific molecular and metabolic events underlying sesame floral development remain largely unclear. In this study, we integrated transcriptomic and metabolomic data across five key developmental stages (T1-T5) to uncover a highly coordinated, temporally regulated network governing floral morphogenesis and maturation.

Joint analyses, including orthogonal partial least squares (O2PLS) and Procrustes analysis, revealed strong concordance between transcriptomic and metabolomic profiles, with gene expression and metabolite accumulation tightly coupled in both space and time. This ensures orderly progression through successive developmental transitions. Pathway analyses further uncovered dynamic shifts in biological priorities across stages. During early development (T1-T2), rapid cell proliferation and primary energy metabolism predominate. Genes involved in fatty acid elongation, glycolysis, and ABC transport are highly expressed at T1, acting synergistically with starch and sucrose metabolism to supply energy and carbon skeletons for intensive cell division (Yang et al., 2019). The middle phase (T2-T4) emerges as a critical window for secondary metabolism activation and defense system establishment. Multi-omics data reveal highly dynamic, coordinated changes in gene expression and metabolite abundance. Early induction of diterpenoid and stilbenoid biosynthesis at T2 signals the onset of chemical defense responses (Del Baño et al., 2003). Concurrently, flavonoid biosynthesis, phenylpropanoid metabolism, and plant–pathogen interaction pathways are continuously enriched, indicating systematic deployment of a phenylpropanoid-centered defense system alongside floral organ differentiation (Choi and Kim, 2022). During late development (T4-T5), regulatory focus shifts toward hormone-mediated organ maturation and terminal metabolite synthesis. Plant hormone signal transduction and isoquinoline alkaloid biosynthesis become dominant pathways, highlighting hormonal control of final floral maturation. Concomitant enrichment of α-linolenic acid, phenylalanine, and Calvin cycle pathways at T5 reflects physiological transitions toward photosynthetic independence and membrane lipid remodeling upon full flower expansion (Muhlemann et al., 2012; Müller et al., 2010). The marked reduction in co-enriched pathways at T4–T5 further indicates developmental completion and progressive metabolic specialization.

The phenylpropanoid/flavonoid pathway exhibits strict temporal control. Upstream genes (*PAL*, *C4H*, *4CL*) are highly expressed at T1 to supply pathway precursors (Fraser and Chapple, 2011). Early *DFR* expression coincides with leucoanthocyanidin accumulation, initiating anthocyanin biosynthesis (Shirley et al., 1992; Winkel-Shirley, 2001). Mid-to-late stages witness sequential activation of pathway branches: *CHS*, *CHI*, and *F3H* are strongly upregulated at T4–T5, driving naringenin chalcone and naringenin synthesis to sustain pollen fertility (Wang et al., 2020). *ANR* expression aligns with epicatechin accumulation, while early-to-mid *UGT* (SIN_1016486/488) expression supports proanthocyanidin synthesis and antioxidant protection (Dixon and Steele, 1999; Menssen et al., 1990; Saigo et al., 2020). Late-stage *UGT* (SIN_1010153) upregulation promotes flavonol and anthocyanin accumulation, enhancing UV tolerance (Henry-Kirk et al., 2018). Flavonols further modulate auxin transport to fine-tune floral patterning (Peer et al., 2004). Stage-specific *UGT* expression precisely governs metabolite glycosylation, modulating stability and biological activity (Dong et al., 2020). Compared with other species, sesame displays a temporally more resolved regulatory cascade. In Arabidopsis, the flavonol branch is primarily governed by SG7 R2R3-MYB transcription factors MYB11, MYB12, and MYB111, whereas SG19 MYBs MYB21, MYB24, and MYB57 specifically drive flavonol accumulation in pollen and stamens (Mehrtens et al., 2005; Stracke et al., 2007), with MYB12 directly activating CHS, CHI, F3H, and FLS. In Brassica napus, anthocyanin structural genes including F3’H, DFR, ANS, and UFGT are regulated by the MBW complex PAP2-TT8-TTG1, and a feedback loop involving the R3-MYB repressor BnCPC fine-tunes anthocyanin levels (Xie et al., 2022). In soybean, GmMYB108, a flower-specific R2R3-MYB transcription factor, acts as a positive regulator of flavonoid and anthocyanin biosynthesis. Despite these advances, most studies compare only two or three developmental stages, leaving the temporal architecture of phenylpropanoid activation largely coarse. In contrast, our five-stage resolution reveals a gene-to-metabolite relay in which upstream phenylpropanoid genes (PAL, C4H) peaked at early stages, downstream flavonoid genes (CHS, CHI, F3H) were sequentially induced at mid-to-late stages, and UGT-mediated glycosylation corresponded with the accumulation of specific flavonoid end-products—a regulatory cascade substantially more detailed than the two-stage patterns reported in rapeseed or the stage-specific shifts documented in soybean. Notably, the division of labor among UGT genes across different developmental windows—early UGTs channeling toward proanthocyanidin synthesis and late UGTs promoting flavonol and anthocyanin accumulation—has not been observed with this level of temporal precision in other oilseed crops, pointing to a distinctive regulatory logic in sesame floral development.

Floral regulatory strategies vary substantially across plant groups. In monocot cereals such as rice, wheat, and maize, florigen signaling plays a central role (Yoshikawa and Boden, 2024). Weak florigen signals promote branching and increase spikelet number at the expense of fertility, whereas strong signals accelerate maturation, forming a trade-off between branching and fertility. Key regulators including *FON*, *LOG*, *SPL*, and *APO1* control meristem activity and organ identity (Yoshida and Nagato, 2011). In model dicots like Arabidopsis and snapdragon, the ABC model governs floral patterning, with *LFY*, *AP1*, and *WUS* specifying meristem identity and CO-GI-FT integrating photoperiod cues (Weigel, 1995; Zahn et al., 2010). Basal eudicots such as Eschscholzia californica exhibit partial conservation alongside divergent A-gene function (Zahn et al., 2010). In dioecious spinach, floral development is regulated by *MIKC-MADS*, *MYB*, and *NAC* transcription factors, with hormones mediating sexual differentiation (Ma et al., 2024). In Arabidopsis, photoperiod differentially regulates phenylpropanoid branches to control flavonoid accumulation without affecting lignin (Leung et al., 2023), while in sesame the photoperiod-responsive gene *SiCOL1* regulates flowering time (Zhou et al., 2018) and CONSTANS-like proteins participate in diverse processes beyond flowering given the pronounced stage-specific activation of the phenylpropanoid/flavonoid pathway during sesame floral development observed in our study, we speculate that photoperiod signals—potentially mediated through SiCOL1—may influence flavonoid and related metabolite accumulation during floral organ maturation. As a typical short-day plant, sesame flowering time is strictly regulated by photoperiod. Its indeterminate inflorescence results in asynchronous floral development at different nodes, and the flowering stage is highly sensitive to environmental stress. Based on the findings of this study, subsequent work could combine short-day treatment to explore whether the phenylpropanoid/flavonoid pathway participates in the regulation of floral development by light signals. To address asynchronous floral development at different positions within the indeterminate inflorescence, single-cell or spatial transcriptomics could be applied to reveal molecular heterogeneity among floral organs at the same developmental stage but different nodes. In addition, the expression stability and function of key genes identified in this study such as *MYB*, *AP2/ERF*, and *UGT* could be validated under abiotic stresses including drought and high temperature. CRISPR-Cas9-mediated editing of these genes combined with metabolomic profiling would help clarify their specific roles in stress tolerance and fertility maintenance during floral development. Meanwhile, future studies employing targeted metabolomics with authentic standards are warranted to precisely quantify the absolute concentrations of key flavonoid and anthocyanin compounds, thereby validating the relative abundance trends revealed in the present study. These efforts will facilitate the translation of the theoretical findings into practical strategies for improving sesame adaptability.

## 5 Conclusion

Sesame flower development is driven by a series of tightly coordinated transcriptional and metabolic transitions, yet the timing and regulatory logic of these events remain largely unknown. To dissect this process, we performed integrated transcriptomic and metabolomic profiling across five consecutive stages, from floral bud initiation to pistil maturation. Our results showed that early development (T1–T2) was dominated by primary carbon metabolism, supporting rapid cell proliferation. A marked transition occurred at T3, where DNA replication genes were downregulated while phenylpropanoid biosynthesis genes were induced, signaling the onset of secondary metabolism. This shift intensified at T4–T5, during which flavonoid and anthocyanin biosynthesis pathways were strongly activated, accompanying the accumulation of pigmented metabolites and the preparation for pollination. Weighted gene co-expression network analysis identified two developmentally relevant modules: one enriched in phenylpropanoid genes and positively correlated with late stages, and another enriched in DNA replication and repair genes and negatively correlated with developmental progression, reflecting a reciprocal relationship between proliferation and metabolic specialization. Within the phenylpropanoid pathway, key enzyme genes—from PAL and C4H to CHS, CHI, F3H, and UGT—were sequentially upregulated, followed by corresponding accumulation of flavonoid end-products, revealing a clear precursor-to-product relay. Collectively, this study not only deepens our understanding of the molecular mechanisms governing reproductive development in sesame but also provides a theoretical foundation and candidate gene resources for improving floral organ traits through molecular breeding.

## Data Availability

The datasets presented in this study can be found in online repositories. The names of the repository/repositories and accession number(s) can be found in the article/**Supplementary Material**.

## Supplementary material

Supplementary Figure 1 Weighted gene co-expression network analysis (WGCNA) of gene expression during sesame flower development. **(A)** Scale-free topology fit index under gradient soft threshold powers. **(B)** Mean gene connectivity corresponding to different soft threshold powers. **(C)** Bar plot showing module significance (MS) of all co-expression modules. **(D)** Scatter plot of module membership (MM, kME) versus gene significance (GS) for the blue module. **(F)** Scatter plot of module membership (MM, kME) versus gene significance (GS) for the turquoise module.

## Glossary

4CL: 4-coumarate-CoA ligase
ABC: ATP-binding cassette
ANR: anthocyanidin reductase
ANS: anthocyanidin synthase
AP2/ERF: APETALA2/ethylene-responsive factor
bHLH: basic helix-loop-helix
C4H: cinnamate 4-hydroxylase
CEN2: centroradialis-like 2
CHI: chalcone isomerase
CHS: chalcone synthase
COG: Clusters of Orthologous Groups
DAM: differentially accumulated metabolite
DEG: differentially expressed gene
DFR: dihydroflavonol 4-reductase
EMS1: EXCESS MICROSPOROCYTES 1
ERF: ethylene-responsive factor
F3’5’H: flavonoid 3’,5’-hydroxylase
F3H: flavanone 3-dioxygenase
FDR: false discovery rate
FLS: flavonol synthase
FPKM: Fragments Per Kilobase of transcript per Million mapped reads
GO: Gene Ontology
GRAS: GAI-RGA-SCR
KEGG: Kyoto Encyclopedia of Genes and Genomes
LC-MS/MS: liquid chromatography-tandem mass spectrometry
MCM: minichromosome maintenance
MYB: myeloblastosis
NCBI: National Center for Biotechnology Information
NGDC: National Genomics Data Center
NR: Non-Redundant
O2PLS: two-way orthogonal partial least squares
OFP: OVATE family protein
PAL: phenylalanine ammonia-lyase
PCA: principal component analysis
QC: quality control
qRT-PCR: quantitative real-time polymerase chain reaction
QTL: quantitative trait locus
RNA-seq: RNA sequencing
RSD: relative standard deviation
SRA: Sequence Read Archive
STEM: Short Time-series Expression Miner
Swiss-Prot: Swiss-Prot protein knowledgebase
TPM: Transcripts Per Million
UGT: UDP-glucosyltransferase
VIP: Variable Importance in Projection
WGCNA: Weighted Gene Co-expression Network Analysis
ZF-HD: zinc finger homeodomain
