# Supplementary material for: Integrated transcriptome and metabolome analyses uncover stage-specific dynamic regulatory networks during sesame floral development
Source: Front Plant Sci. 2026 Aug 13;17:1918255. doi: 10.3389/fpls.2026.1918255 (PMC13518558; doi:10.3389/fpls.2026.1918255)
Supplement: Supplementary file 1 [file Image1.pdf]

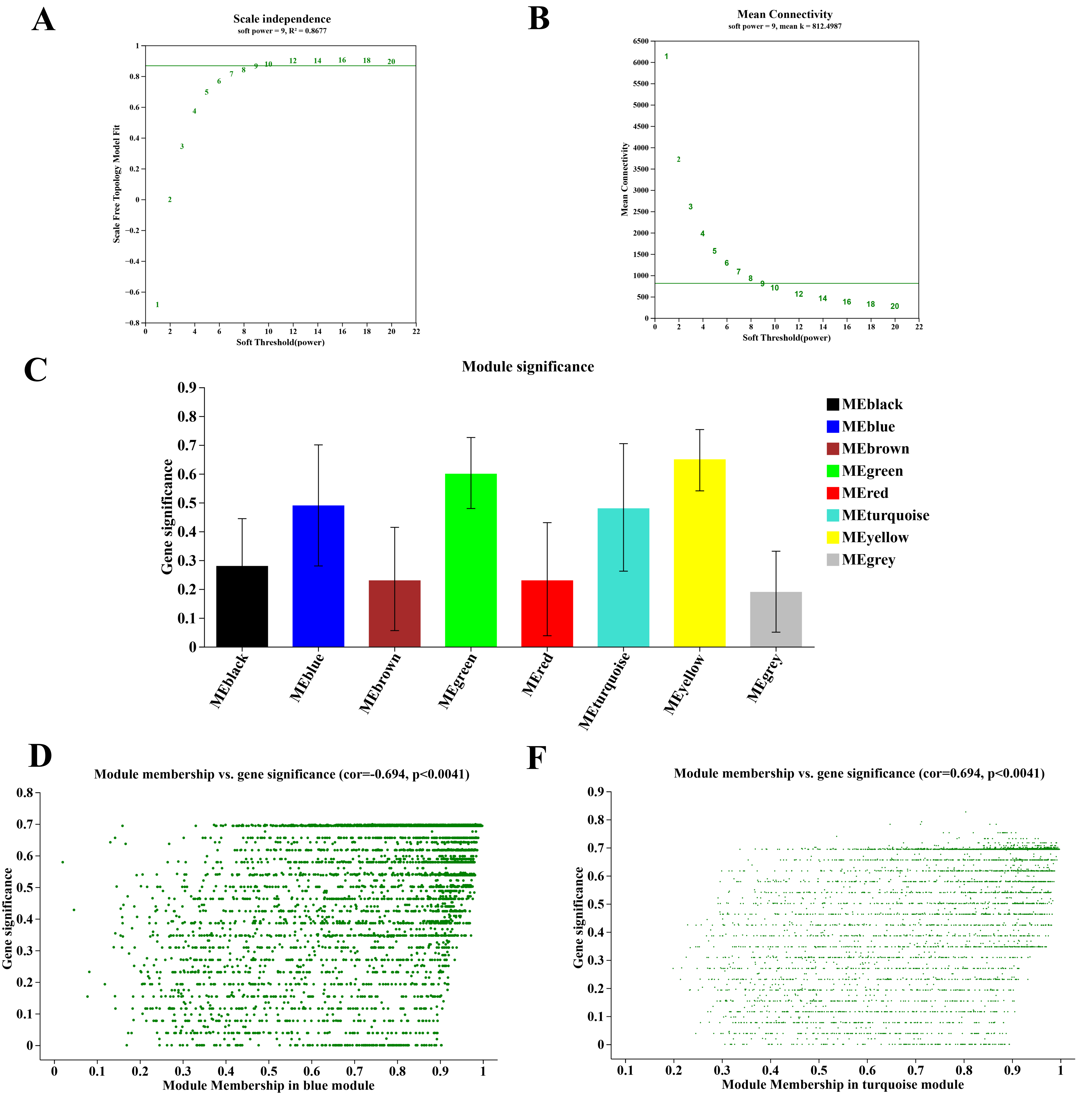

A
Scale independence
soft power = 9, R² = 0.8677
Scale Free Topology Model Fit
Soft Threshold(power)
B
Mean Connectivity
soft power = 9, mean k = 812.4987
Mean Connectivity
Soft Threshold(power)
C
Module significance
Gene significance
MEblack
MEblue
MEbrown
MEgreen
MEred
MEturquoise
MEyellow
MEgrey
D
Module membership vs. gene significance (cor=-0.694, p<0.0041)
Gene significance
Module Membership in blue module
F
Module membership vs. gene significance (cor=0.694, p<0.0041)
Gene significance
Module Membership in turquoise module
